# Attitude towards gender roles and violence against women and girls (VAWG): baseline findings from an RCT of 1752 youths in Pakistan

**DOI:** 10.1080/16549716.2017.1342454

**Published:** 2017-07-31

**Authors:** Tazeen Saeed Ali, Rozina Karmaliani, Judith Mcfarlane, Hussain M. A. Khuwaja, Yasmeen Somani, Esnat D. Chirwa, Rachel Jewkes

**Affiliations:** ^a^ School of Nursing and Midwifery and Department of Community Health Sciences, Aga Khan University , Karachi, Pakistan; ^b^ Health Policy & Management, School of Nursing and Midwifery and Department of Community health sciences, Aga Khan University , Karachi, Pakistan; ^c^ College of Nursing, Office of Research and Sponsored Programs, Texas Women’s University , Houston, TX, USA; ^d^ Health Policy and Management, Aga Khan University , Karachi, Pakistan; ^e^ School of Nursing and Midwifery, Aga Khan University , Karachi, Pakistan; ^f^ Gender & Health Research Unit, South African Medical Research Council , Pretoria, South Africa; ^g^ Gender & Health Research Unit, South African Medical Research Council , Pretoria, South Africa

**Keywords:** Gender roles, gender norms, youth perpetration, youth victimization, violence against women and girls (VAWG)

## Abstract

**Background**: Violence against women is driven by gender norms that normalize and justify gender inequality and violence. Gender norms are substantially shaped during adolescence. Programs offered through schools offer an opportunity to influence gender attitudes toward gender equity if we understand these to be partly shaped by peers and the school environment.

**Objective**: We present an analysis of the baseline research conducted for a randomized controlled trial with 1752 grade 6 boys and girls and their attitudes toward gender roles, VAWG, and associated factors.

**Methods**: We used baseline data from a  cluster randomised control study. Interviews were conducted in 40 public schools in Hyderabad, with 25–65 children per school. Questions were asked about attitudes toward gender roles, peer-to-peer perpetration, and victimization experiences, and family life, including father- or in-law-to- mother violence and food security. Multiple regression models were built of factors associated with gender attitudes for boys and girls.

**Results**: Our result have shown youth attitudes endorsing patriarchal gender beliefs were higher for boys, compared to girls. The multiple regression model showed that for boys, patriarchal gender attitudes were positively associated with hunger, depression, being promised already in marriage, and being a victim and/or perpetrator of peer violence. For girls gender attitudes were associated with hunger, experiencing corporal punishment at home, and being a perpetrator (for some, and victim) of peer violence.

**Conclusion**: Youth patriarchal attitudes are closely related to their experience of violence at school and for girl’s physical punishment, at home and for boys being promised in early marriage. We suggest that these variables are indicators of gender norms among peers and in the family. The significance of peer norms is that it provides the possibility that school-based interventions which work with school peers have the potential to positively impact youth patriarchal gender attitudes and foster attitudes of gender equality and respect, and potentially to decrease youth victimization and perpetration.

## Background

Violence against women occurs and continues, in a large part, because of gender norms occurring at the societal and family levels. Patriarchal gender norms and values reinforce and sustain the low status of girls and women in society and increase the likelihood that boys and men will perpetrate violence against girls and women. Gender attitudes and roles are usually learned in childhood and research documents that peer group norms regarding acceptable masculine and feminine behaviors and gender values are incorporated into self-concept as strongly as role modeling observed in the nuclear family [1]. Research also documents that gender inequity and early subordination of females places adult females in greater risk of intimate partner violence [2]. If gender inequitable attitudes are held and roles imposed or embraced in adolescence, adult women are at greater risk of intimate partner violence. According to Kagesten systematic review of literature on factors that shape gender attitudes in early adolescence across different cultural settings globally found 82 articles but 90% of these were from North America and Western Europe [3]. There were three papers from South Asia, India, and none from Pakistan. It was found that young adolescents commonly voice gender inequitable attitudes, with some variation by social and demographic characteristics. It was found that influences of key groups in adolescents’ lives including family and peers are central in young adolescents’ construction of gender attitudes. Further, that gender socialization processes differed for boys and girls [3].

Gender inequity is common in Pakistan because of the prevalence of conservative cultural traditions based on male superiority and entitlement [2,4]. Society reinforces gender inequity in Pakistan with over half of the adult women illiterate. Young girls are raised to tolerate and accept intimate partner violence (IPV) as a part of life in Pakistan, with United Nations statistics 2015 reporting over 50% of teenage girls believed that violence enacted upon them is justified [5]. Furthermore, misinterpretation of religion is often used to justify intimate partner violence (IPV) [6]. Patriarchal gender norms and associated gender inequity result in high rates of IPV in Pakistan, with over 70% of wives reporting abuse [6]. Recently, ‘street macho’ has been described as the attitude of masculinity amongst the male youth of Pakistan, even among those from educated backgrounds. This street macho is reported as many young men feeling that it is acceptable to oppress women. These same young men embrace a violent masculinity, that draws strength from the aura of power that they perceive emanates from men who abuse women [7]. Clearly, social and cultural norms can create a climate in which violence against girls and women is encouraged and normalized.

Peer youth victimization and perpetration is a global epidemic that intersects with patriarchal gender attitudes to accentuate youth violence. The report from the World Health Organization (WHO) on preventing youth violence calls for strategies to strengthen norms and values that support non-violent, respectful, nurturing, positive, and gender equitable relationships among children and adolescents [2]. Strategies are needed to modify deeply ingrained societal and cultural norms and behaviors, especially the notion that some forms of violence, such as beating girls and women, is normal and justified.

To learn what works to prevent youth violence in Pakistan an evaluation is being conducted in urban public schools of a two-year long intervention delivered by the NGO Right to Play which used the transformative power of sport to empower children and build more equitable and non-violent attitudes [8]. This paper draws on the baseline data from the evaluation and describes gender attitudes and the factors associated with holding more patriarchal attitudes among 1752 school-going boys and girls of Grade 6 in Pakistan. The model which guided the analysis started from the premise that gender attitudes of boys and girls are likely to be influenced by factors in the ecological context of their lives. These include socio-demographic factors and individual mental health (indicated by depressive symptoms), gender attitudes at home (expressed through the use of violence at home which we connect to masculinity of the father), and gender attitudes of peers and other schooling factors.

## Methods

This paper is based on an analysis of the baseline data collected as part of a cluster randomized controlled trial. Further details of the trial are described elsewhere [9]. Following Ethics Review Approval (Aga Khan University Review Document number 3705), data were collected in 40 single gender government schools (20 for each gender) in the city of Hyderabad over a time period of 60 days. Hyderabad is located in the southern province of Sindh, at about 160 km from the large city of Karachi. Recruitment was done in the sixth grade of 12–14 years of age after consent was obtained from their parents and themselves. The children needed to understand the national (Urdu) or provincial (Sindhi) language so they could self-complete the questionnaire which was read to them in small groups by a trained data collector. Among the children who returned the signed parental consent, 95% of the youth offered their assent. A total of 1752 children filled the self-reported instruments

### Variables in the instruments

Gender attitudes were measured in a 12-item, investigator derived measure that assessed child agreement on a Likert scale (i.e., Strongly agree = 1, Agree = 2, Disagree = 3, and Strongly disagree = 4) to a series of statements. Items include girls going to school, wives having a say in family finances, husband’s right to punish wives, and women’s participation in social events and employment (items are shown in Table 1). The 12 items were summed into a composite score that ranged from 12 to 48. Higher values on this scale represented greater disagreement with aspects of women’s autonomy and participation in society, which we term patriarchal gender attitudes. Coefficient alpha for this variable were 0.763 (boys) and 0.821(girls).

**Table 1.** Summary of gender attitudes items by Gender.

		Boys		Girls		
Item		n	%	n	%	p-value
I think girls in my family should go to school	Strongly agree	589	71.7	755	81.2	<0.001
		Agree Disagree/strongly disagree	159	19.3	154	16.6	
		74	9.0	21	2.3	
I think my father should give to permission to my mother to go the clinic.	Strongly agree	519	63.1	678	72.9	<0.001
		Agree	165	20.1	174	18.7	
		Disagree/strongly disagree	138	16.8	78	8.4	
I think my father should listen to my mother’s opinion on schooling	Strongly agree	593	72.1	721	77.5	0.001
		Agree	156	19.0	177	19.0	
		Disagree/strongly disagree	73	8.9	32	3.4	
I think my mother should have a say in how money is spent in the family	Strongly agree	536	65.2	671	72.2	0.024
		Agree	198	24.1	177	19.0	
		Disagree/strongly disagree	88	10.7	82	8.8	
I think my mother should be able to ask a religious scholar about the solution of issues	Strongly agree	494	60.1	586	63.0	0.139
		Agree	193	23.5	184	19.8	
		Disagree/strongly disagree	135	16.4	160	17.2	
I think my father should respect the opinion of my mother on matters related to income-generating work	Strongly agree	587	71.4	710	76.3	0.003
		Agree	173	21.0	182	19.6	
		Disagree/strongly disagree	62	7.5	38	4.1	
I think my father should be kind and caring towards women in my family	Strongly agree	635	77.3	765	82.3	0.026
		Agree	143	17.4	140	15.1	
		Disagree/strongly disagree	44	5.4	25	2.7	
I think that my mother should be always obey my father	Strongly agree	593	72.1	746	80.2	0.001
		Agree Disagree/strongly disagree	168	20.4	148	15.9	
		61	7.4	36	3.9	
I think women should be able to participate in weddings	Strongly agree	563	68.6	680	73.3	<0.001
		Agree Disagree/strongly disagree	182	22.2	210	22.6	
		76	9.3	38	4.1	
I think women should be able to participate in neighborhood events	Strongly agree	511	62.5	656	70.8	<0.001
		Agree	215	26.3	214	23.1	
		Disagree/strongly disagree	92	11.2	57	6.1	
I think women should be able to participate in skills training	Strongly agree	521	63.5	703	75.8	<0.001
		Agree Disagree/strongly disagree	187	22.8	172	18.6	
		112	13.7	52	5.6	
I think women should be able to participate in income-generating activities.	Strongly agree	325	39.7	526	56.6	<0.001
		Agree	164	20	186	20	
		Disagree/strongly disagree	330	40.3	218	23.4	

Peer violence is measured with the Peer Victimization Scale (PVS), a 16-item measure with four subscales, each with four questions, assessing physical and verbal victimization, social manipulation, and property attacks [10]. Respondents were asked over the last four weeks, how often (i.e., never, once, a few times (2–3) or many times (4 or more) an act happened to them (i.e. victimization). Responses were scored (range of 0 to 48). An example of peer victimization is ‘hurt me physically’. Coefficient alpha for this variable was 0.862 (boys) and 0.861 (girls). Peer violence perpetration was measured on a scale which was designed as a mirror image of the victimization scale. An example of peer perpetration is ‘Tripped another child to make him or her fall’. Coefficient alpha for this variable was 0.886 (boys) and 0.864 (girls).

A childhood hunger index was created from two items that asked children how often in the last -four weeks they went to school without breakfast and how often they went to sleep without dinner because of lack of food at home. The hunger index was measured with four-point response categories from ‘never’ to ‘all or most days’. The two items were summed to create a score with a maximum of 8 points.

Child marriage preparation for the child interviewed and in the family was captured by three items asking if the child is promised for marriage, if there have been marriage preparations made, and if female siblings or cousins of a similar age are married.

School performance is measured by asking about performance in the four courses including language, math, science, and social studies. Response categories were measured through the scale of three (fail, average, and excellent). Data is also collected about the number of days of school missed in the last four weeks with its main reason, including whether the child is working at home or for money outside of home.

We measured depression with the Child Depression Inventory II (CDI-2) [11]. This has not been validated in Pakistan. However, it was not used for diagnostic purpose but to measure symptoms and there is no reason to expect it to perform very differently in Pakistan from the many settings in which it has been more formally tested. This is a 28-item self-report questionnaire designed to assess the severity of current or recent depressive symptoms in children aged 7 to 17. The CDI-2 items are rated on a 3-point scale as 0 = no symptom, 1 = probable or mild symptom, and 2 = definite, marked symptom. A CDI-2 score was derived from the 28-items with higher scores representing increased depressive symptoms. Coefficient alpha for this study were 0.723 (boys) and 0.719 (girls). For this study, the raw scores for the CDI-2 were converted into t-scores based on age and gender attributes of the participants according to the specifications in the CDI-2 technical manual [12]. The T-scores range from ≤40 to ≥90, and a score of 65 or more is considered by Kovacs as indicative of a high level of depressive symptomatology which may indicate depression.

In order to provide indicators of gender norms and use of violence at home we asked about violence used against the child’s mother in the last four weeks: seeing or hearing father hitting mother, and seeing or hearing mother being beaten by other relatives. These items were measured on four point scale (0 = never, 1 = once, 2 = few times, 3 = many times), which was later transformed into dichotomous (ever/never) items. We also asked about whether the child had seen or heard the father having a physical fight with other men in the last four weeks.

To measure use of physical punishment we asked if the child have been ‘slapped, beaten, or otherwise physically punished by a parent and whether they had been beaten so hard that they got injured.

### Data analysis

All analysis took into account the study design, which is a cluster randomized control design, with participants clustered within schools. All potential explanatory variables of gender attitudes were summarized by gender of participant. Summary statistics for categorical explanatory variables were frequencies and percentages, while means and standard deviations were used as summary statistics for continuous variables. Chi-square tests were used to compare gender attitudes between boys and girls, using the standard linearization methods for clustered data and a t-test for the comparison of means.

Generalized linear mixed effect models were used to assess the bivariate relationships between possible explanatory variables and gender attitude score for boys and girls. Prior to modeling factors associated with gender attitudes, the distribution of the gender attitude score was assessed for normality.

Generalized linear multivariate model was used to present factors associated with gender attitudes amongst learners, with school as a random effect. Gauss-Hermite quadrature integration method was used to obtain likelihood functions [13]. The multiple regression models were built in groups. The first model looked at household factors and violent behavior and gender norms factors as explanatory variable. The second model explored the schooling factors that are associated with gender attitudes, while the third model examined the relationship between gender attitude and depression subscales. Backward elimination was used to determine associations in each group (up to p = 0.15), and only factors that were associated at this level in the bivariate analysis were considered for the multiple regression. The final model combined more strongly associated factors from the three models, using the backward elimination method to get the final model. The inclusion criteria initially for the backward elimination was a p-value of 0.15, as suggested by Vittinghoff et al. [14]. Only factors with p-value less than 5% were retained in the final model. Model diagnostics (using deviance residuals) were performed on the final model to examine normality and also check for lack of fit. All analyses were done using Stata 14 software package.

## Results

The mean age for boys was 12.5, (SD 1.5) and mean age for girls was12.3 (SD 1.38) (Table 2).Boys experienced higher levels of hunger (as shown on the hunger index) compared to girls, with the mean for boys 0.65 (SD 1.09), and for girls 0.48 (SD 0.97). The proportion of boys who did not always have breakfast before school as there was no food at home was 24%, compared to 18% of girls respectively.

**Table 2.** Descriptive statistics by gender of participant.

	BOYS(N = 822)			GIRLS(N = 930)			
Factor	N	n or mean	% or sd	N	n or mean	% or sd	p-value
Age of learner**^a^**	821	12.51	1.5	927	12.27	1.38	0.001
Days missed school**^a^**	813	4.07	4.17	927	3.14	2.85	0.001
School performance**^a^**	822	9.27	1.81	930	9.55	1.77	0.065
Hunger index**^a^**	822	0.65	1.09	930	0.48	0.97	0.016
**Peer violence experience/perpetration**	822			930			
None		55	6.7		194	20.9	<0.001
Victim only		146	17.8		265	28.5	
Any perpetration		621	75.5		471	50.6	
**In the previous 4 weeks:**							
Experienced physical punishment at home	821	495	60.3	930	345	37.1	<0.001
Missed school due work at home	795	195	24.5	917	130	14.2	0.005
Missed school due to working for money	796	61	7.7	916	15	1.6	<0.001
Father fought with other men	820	211	25.7	928	166	17.9	0.002
Father beat mother	822	80	9.7	929	56	6	0.016
Relatives hit mother	821	38	4.6	929	36	3.9	0.396
Father drunk	818	28	3.4	926	25	2.7	0.350
**Child marriage:**							
Promised in marriage	819	52	6.3	927	25	2.7	0.012
Other preparations made for marriage	820	19	2.3	928	23	2.5	0.766
Sister/cousin of same age married	814	38	4.7	924	73	7.9	0.018
Depression (high = more depressed)**^a^**	817	56.75	9.69	921	54.61	9.26	0.002
Gender attitude score(high = inequitable)**^a^**	812	20.76	4.70	923	19.14	4.56	<0.001

**^a^** Summary stats for continuous variables represented as mean and standard deviation.

School attendance was irregular for many boys and girls in the last four weeks, where the average number of days missed from schools for boys was 4.1 (SD 4.2) and for girls was 3.1 (SD 2.8). The mean self-rated school performance for boys (9.2) was lower than girls (9.5), and the proportion of children reporting the main reason for their last day missed from school as due to work at home was 24.5% for boys and 14.2% for girls. The proportion of adolescents that missed school in last four weeks to earn money was 7.7% for boys and 1.6% for girls.

Overall engagement in peer violence in the prior four weeks was common with the proportion only having experience of victimization among boys was 17.8% and among girls, 28.5%. In addition, 75.5% of boys and 50.6% of girls had been a perpetrator of violence and many of these were victims as well. The proportion of boys and girls who had experienced physical punishment at home in the previous four weeks was 60.3% and 37.1% respectively. The proportion of children who observed their father fight at home in the last four weeks was very high (25.7% of boys and 17.7% of girls). Many of them had also witnessed violence against their mother by their father (9.1% boys and 6.0% girls), or another relative (3.4% of boys and 3.9% of girls). The measure of depression showed boys scoring somewhat higher than girls, with a mean of 56.8 versus 54.6.

Boys scored more highly than girls on the measure of gender attitudes, with the mean for boys 20.8 and for girls 19.1. A small proportion of the children had already been promised in marriage and this was less common for girls (2.7%) than boys (6.3%). For most of these girls (n = 23/35) preparation had already started for the marriage, whereas this was only reported for 38% of the promised boys (n = 19/50). Girls more commonly reported that they had a similar aged sister or cousin (7.9% of girls and 4.7% of boys) who had been married.

Frequencies and percentages for the items of the gender attitudes scale, stratified by gender, are presented in Table 1. The direction of scoring is such that higher values signify more patriarchal gender attitudes. There was a significant difference in gender attitudes between boys and girls on all but one question. Girls were in greater disagreement with aspects of patriarchal gender attitudes than boys. For example, 81.2% of girls strongly agreed that girls in their family should go to school, whereas for boys only 71.7% strongly agreed. However, only one item ‘I think my mother should be able to ask a religious scholar about the solution of issues’ was not different and 60.1% of boys and 63. 0% of girls agreed with the question.

Table 3 shows bivariate analyses of associations between variables and gender attitudes among boys and girls. For both genders, the hunger and depression scores were significantly associated with patriarchal gender attitudes as was the number of days missed from school for girls.

**Table 3.** Bivariable analysis of factors associated with of gender attitudes among boys and girls.

	BOYS				GIRLS			
	Coef.	LCL	UCL	p-value	Coef.	LCL	UCL	p-value
Age of learner**^a^**	−0.12	−0.33	0.09	0.268	−0.23	−0.45	−0.02	0.034
Hunger index**^a^**	0.64	0.35	0.94	<0.001	0.68	0.38	0.98	<0.001
Depression **^a^**	0.06	0.03	0.09	0.002	0.06	0.03	0.09	<0.001
Days missed school**^a^**	0.08	−0.01	0.16	0.061	0.12	0.02	0.22	0.019
School performance**^a^**	0.07	−0.11	0.24	0.478	−0.06	−0.22	0.11	0.511
**Peer violence experience/perpetration**								
None								
Victim only	2.23	0.82	3.64	0.002	0.73	−0.11	1.56	0.087
Any perpetration	3.33	2.08	4.59	<0.001	1.89	1.14	2.65	<0.001
**In previous 4 weeks**								
Experienced physical punishment at home	1.12	0.47	1.77	0.001	1.26	0.66	1.86	<0.001
Missed school due to work at home	−0.10	−0.87	0.67	0.800	0.01	−0.83	0.84	0.987
Missed school due to work for money	−0.25	−1.48	0.98	0.690	0.78	−1.51	3.06	0.506
Father fought with other men	0.90	0.17	1.63	0.015	0.16	−0.60	0.92	0.674
Father beats mother	0.92	−0.17	2.00	0.098	1.23	0.01	2.45	0.026
Relatives hit mother	2.29	0.79	3.79	0.003	0.86	−0.64	2.36	0.260
Father drunk	0.93	−0.86	2.72	0.307	−2.30	−4.09	−0.52	0.011
**Child marriage**								
Promised in marriage	1.48	0.17	2.80	0.027	0.13	−1.66	1.93	0.885
Other preparation made for marriage	−0.71	−2.93	1.52	0.534	0.55	−1.37	2.46	0.576
Sister/cousin of same age is married.	1.22	−0.33	2.78	0.123	0.57	−0.53	1.68	0.309

NB: All analyses adjusted for clustering by school (random component). ^a^Continuous variables. UCL: upper confidence limit; LCL: lower confidence limit.

The patriarchal gender attitudes score was associated with children reporting experiences of violence for boys only and any perpetration (with/without victimization) for both boys and girls. Similarly, there were associations with aspects of experience of violence at home, including having experienced physical punishment (boys and girls), witnessing their father beat their mother (girls only), witnessing relatives hitting their mother (boys only) and being promised in marriage (boys only).

Table 4 shows results from a multivariable generalized linear mixed model of factors associated with gender attitude scores among boys and girls This shows that for both genders having more patriarchal gender attitudes towards women was associated with having more experience of hunger (boys p value = 0.002 and girls p value = < 0.001), and perpetrating peer violence (boys p value = 0.002 and girls p value = < 0.001). In the case of boys, experiencing peer violence is significantly associated with gender attitudes, but not for girls. Experiencing physical punishment at home was significantly associated in girls but not boys (girls p value = 0.043). Being promised in marriage and depression were positively associated to patriarchal gender attitude for boys only (p value = 0.039; and p-value = 0.047, respectively).

**Table 4.** Multivariable regression models of factors associated with gender attitudes among boys and girls adjusted for age.

	BOYS				GIRLS			
	Coef.	LCL	UCL	p-value	Coef.	LCL	UCL	p-value
Hunger index**^a^** ^/^	0.48	0.18	0.78	0.002	0.55	0.25	0.85	<0.001
**Peer violence experience/perpetration**								
None	Ref				Ref			
Victim only	2.16	0.76	3.57	0.003	0.57	−0.25	1.40	0.174
Any perpetration	2.88	1.61	4.15	<0.001	1.44	0.65	2.22	<0.001
Experienced physical punishment at home					0.65	0.02	1.28	0.043
Promised in marriage	1.36	0.07	2.66	0.039				
Depression score	0.04	0.01	0.07	0.047				

NB: All models adjusted for age of the learner and clustering by school (random component). UCL: upper confidence limit; LCL: lower confidence limit.

## Discussion

This sample of 1752 sixth grade students in Pakistani public schools demonstrates several key differences between gender attitudes of boys and girls and the relationship to violence exposure at home and among peers. Additionally, data is presented on hunger, depression, and early marriage plans. Gender attitudes of youth can potentially contribute to our understanding of root causes of violence against women. The overall findings that boys report significantly higher levels of patriarchal gender attitudes compared to girls is similar to those from other studies conducted in Egypt [15], USA [16], and Brazil [17].

The association between patriarchal attitudes and violent behavior among adolescents has been previously observed [18]. Some research suggests that patriarchal attitudes may lead to violence, as was found amongst grade 8 children in South Africa [19], and this was also supported by structural models of a pathway from food insecurity to peer violence in this dataset [20]. Research from elsewhere shows that experience of victimization decreases empathy towards others and contributes to individual behavior moving from victimization to perpetration [21,22]. This transformative process from victimization to perpetration is most pronounced during adolescence [23]. A recent review found that male peer groups enforce competition, toughness, and heterosexual prowess. Boys who fail to achieve local masculinity standards are bullied and ridiculed by their peers [3].

Perhaps peer violence engagement is operating in our model as an indicator variable for peer group gender attitudes, and it is the peer group attitudes which drive youth violence. This may explain why the association between violence and gender attitude is most pronounced in both genders for perpetration. Among girls there are many girls who are only victimized and may not be part of perpetuating peer violence, whereas for boys, the group that is only victimized is very small. Boys who are victimized are also perpetrators on some occasions and hence these boys may be more similar to the perpetrating group than the report from the last four weeks suggests. This pattern for males has been supported by other literature [19].

Similarly, for both boys and girls, higher levels of food insecurity (i.e., hunger, which is indicative of poverty) is associated with more patriarchal gender attitudes. Research among Pakistani adults and South African children shows an association between poverty and more patriarchal gender attitudes [19,24]. This may be an indication of the gender norms that prevail in more impoverished communities that in multiple settings have often been described as highly patriarchal [25,26]. It is also possible that the patriarchal endorsement of women’s limited participation in income generating activities, endorsed by more than a third of boys and nearly a quarter of girls in our study, reflects ideas from the home and impacts on food availability in the home.

For boys, there was a significant relationship between symptoms of childhood depression and patriarchal gender attitudes. Our findings are similar to the study conducted in South Africa where older adolescent and young adult men with more highly gender inequitable attitudes show more depressive symptomatology [27]. We have not found any study in Pakistan which aimed to explore association between childhood depression and patriarchal gender attitudes.

The variables measuring the violence at home against the child’s mother, father engaging in fights with other men, physical punishment of the child, and questions related to child marriage are indicators of the social environment within which the child lives with respect to gender relations and the use of violence. On all indicators apart from whether a cousin/sibling of a similar age was married, boys report more violent and patriarchal homes compare to girls. The bivariate analysis shows that these variables are associated with more patriarchal attitudes in boys and girls and thus there is a strong correlation between indicators of gender relations at home and use of violence, and children having patriarchal attitudes. However, in the adjusted model only one indicator of patriarchy at home, whether they had been promised in marriage, was associated with gender attitudes in boys. For girls, being beaten at home was associated with more patriarchal attitudes. It may be that beaten at home is an indicator of patriarchy in the Pakistan setting as girls report much less corporal punishment at home than boys [28].

Although significantly more boys report being promised in marriage compared to girls, the girls report significantly more same-age sisters or cousins who are married compared to boys. It is important to note, of those sisters or cousins who are married their median age when married was 16 years for boy participants and 14 years for girl participants. Although more boys report being promised for marriage, girls report more actual marriages of sisters or cousins and at a significantly younger age of 14 compared to male marriage reports at age 16. The global problem of child brides is well documented in the literature [29,30] and the young median age of 14 for girl brides in this study offers further documentation; however, child marriage information did not predict patriarchal gender attitudes scores for girls.

### Limitations

Our research methodology has limitations that it may under- or over-represent youth attitudes toward gender norms and women’s participation, youth-to-youth victimization and perpetration as well as characteristics of family life and youth academic performance. The questions may miss some episodes of youth victimization or perpetration and incorrectly classify others, particularly with respect to the four-week reporting period. Children may not accurately recall the timing and type of victimization or perpetration they experienced (i.e., whether or not the exposure occurred within the last four weeks). The researchers acknowledge recall bias in all questions. With cross-sectional data it is hard to know if a relationship is causal, but in this case it’s likely to be bi-directional, meaning patriarchal gender attitudes may influence peer violence, and engagement in peer violence may directly impact gender attitudes as it is possible that victimized youth tend to gravitate towards more restrictive and patriarchal attitudes [20,31]. Finally, our participants were limited to Sindhi and Urdu speakers, although these are the languages of teaching in the participating schools. Despite these limitations, the researchers feel this study provides a framework for examining youth attitudes toward gender norms within the context of youth-to-youth violence, family life characteristics, and youth academic performance.

## Conclusions

This is the first study on gender attitudes in early adolescence in Pakistan and from the literature review it appears that it is the first to look at factors such as hunger, engagement in peer violence, corporal punishment at home, and depression as associated factors. Research on social norms and gender attitudes shows that these are generally influenced by both peers and family behavior [3]. Our analysis supports that peer violence is associated with patriarchal gender attitudes. Moreover, child marriage and corporal punishment are indicative of the gender attitude in the home. This is an important finding as it raises the possibility that an intervention targeting children in school might impact gender attitudes and norms in school, and at home. Further, since violence perpetration among boys is associated with higher levels of patriarchal gender attitudes, a community based intervention might be necessary, which addresses the patriarchal gender attitudes to potentially decrease youth violence perpetration and victimization. Moreover, youth violence perpetration is also associated with adult violence perpetration and so the potential for a long-term impact is raised [32]. Gender attitudes are important as they form gender norms, and research has shown that women who live in areas with most inequitable gender norms are most likely to experience partner violence victimization [33]. Women’s risk of violence is particularly high when women and their partners share beliefs about the acceptability of violence [34]. There is a need to develop and implement comprehensive effective strategies at the youth level to potentially prevent the occurrence of violence against women and girls.

## Paper context

Gender inequality drives violence against women and girls through gender norms established in childhood. Among Pakistani grade 6 children, poverty and depression drive patriarchal gender attitudes, alongside gender norms at home and among peers, as shown by the links to youth violence. Interventions are needed in schools to empower girls and boys to challenge prevailing gender norms, such as the intervention of the NGO Right To Play. It is very important that we understand its impact.
